# Monolayer Twisted Graphene-Based Schottky Transistor

**DOI:** 10.3390/ma14154109

**Published:** 2021-07-23

**Authors:** Ramin Ahmadi, Mohammad Taghi Ahmadi, Seyed Saeid Rahimian Koloor, Michal Petrů

**Affiliations:** 1 Nano-Physics Group, Nano-Technology Research Center, Physics Department, Faculty of Science, Urmia University, Urmia 5756151818, Iran; ahmadiramin92@gmail.com; 2 Institute for Nanomaterials, Advanced Technologies and Innovation (CXI), Technical University of Liberec (TUL), Studentska 2, 461-17 Liberec, Czech Republic; 3 Technical University of Liberec (TUL), Studentska 2, 461-17 Liberec, Czech Republic; michal.petru@tul.cz

**Keywords:** diameter, geometry characteristic, quantum tunneling, schottky transistor, twisted graphene

## Abstract

The outstanding properties of graphene-based components, such as twisted graphene, motivates nanoelectronic researchers to focus on their applications in device technology. Twisted graphene as a new class of graphene structures is investigated in the platform of transistor application in this research study. Therefore, its geometry effect on Schottky transistor operation is analyzed and the relationship between the diameter of twist and number of twists are explored. A metal–semiconductor–metal twisted graphene-based junction as a Schottky transistor is considered. By employing the dispersion relation and quantum tunneling the variation of transistor performance under channel length, the diameter of twisted graphene, and the number of twists deviation are studied. The results show that twisted graphene with a smaller diameter affects the efficiency of twisted graphene-based Schottky transistors. Additionally, as another main characteristic, the I_D_-V_GS_ is explored, which indicates that the threshold voltage is increased by diameter and number of twists in this type of transistor.

## 1. Introduction

**Figure 1:**
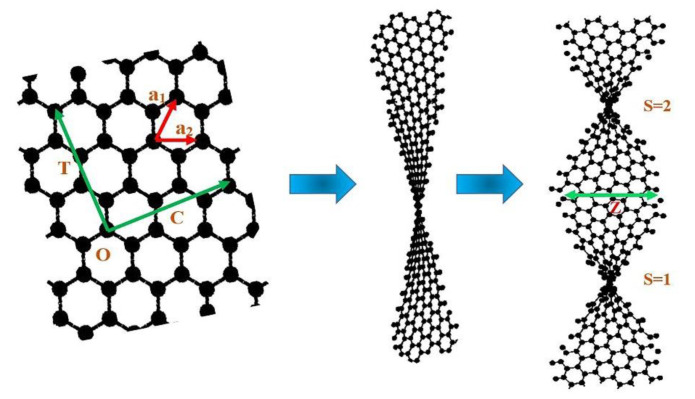
The twisted graphene that is achieved from a graphene sheet.

In the last decade, most efforts of nanotechnology researchers have been devoted to the study and characterization of electronic properties of graphene [1,2,3,4,5,6,7]. Indeed, graphene is a two-dimensional honeycomb structure with high electron mobility and stable lattice which has different classifications [8,9,10,11], such as graphene nanoscroll, twisted graphene, graphene nanoribbon, and few-layer graphene [12,13,14,15,16,17]. Among these different types of graphene, twisted graphene is a new class and interesting [18]. Figure 1 shows how the graphene twist is formed from graphene. Different methods, such as stacking of two single-layer graphenes (SLG) [19], cutting-rotation-stacking (CRS) technique of SLG [20], control folding of SLG [21], selective pick-up and transfer of SLG [22], CVD using decaborane on Copper foil [23] and joule heating of polyaromatic hydrocarbons (PAHs) on Nickel foil [24], have been reported for the synthesis of twisted graphene. Edge shapes of twisted graphene are divided into two types: either zigzag or armchair, and each type has a significant role in electronic properties, which are identified by pair of (*n*, *m*). The *n* and *m* are important coefficients in the chiral vector.

In the zigzag edge, the pairs are converted to (*n*, 0). Based on this theory, (22, 0), (28, 0) and (34, 0) are metallic, since (*n* − 1) is a multiple of three, and hence (30, 0), (39, 0) and (45, 0) are semiconducting. These different features of twisted graphene lead to great attention from researchers.

**Figure 2:**
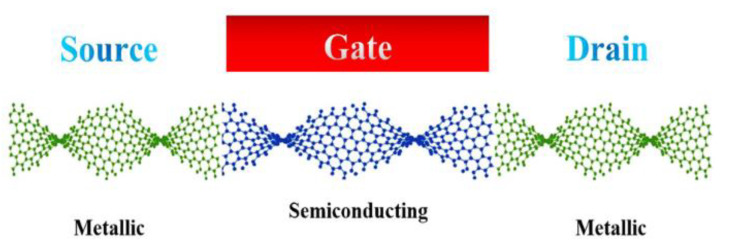
The proposed model.

Considering this variety of properties, twisted graphene can be used in different areas of nanoelectronic components such as diodes and transistors. In the present study, a Schottky transistor based on twisted graphene is modeled. Figure 2 indicates the schematic design of the proposed transistor by assuming metallic and semiconducting twisted graphene. The geometry of twisted graphene is analytically modeled. Essential characteristics for the performance of transistors are also discussed.

## 2. Analytical Modeling

To investigate the electronic properties of twisted graphene, the dispersion relation based on the geometrical effect of twisted graphene should be studied. The graphene sheet has two perpendicular vectors called translation (*T*) and chiral (*C*) vectors as shown in Figure 1. *T* and *C* vectors are defined as:

(1) $$\vec{C}=n\vec{a_{1}}+m\vec{a_{2}}=(n,m)$$

and

(2) $$\vec{T}=t_{1}\vec{a_{1}}+t_{2}\vec{a_{2}}=(t_{1},t_{2})$$

where *a*_1_ and *a*_2_ are the basic vectors:

(3) $$\left(\vec{a_{1}}=\sqrt{3}a_{c-c}(\frac{\sqrt{3}}{2}\vec{i}+0.5\vec{j}\right)\mathrm{and}\vec{a_{2}}=\sqrt{3}a_{c-c}(\frac{\sqrt{3}}{2}\vec{i}-0.5\vec{j})$$

The *n* and *m* are integer numbers and *a*_c-c_ is the *C-C* bond length. The *t*_1_ and *t*_2_ are obtained based on the perpendicular property of *T* and *C*:

(4) $$t_{1}=\frac{2m+n}{d_{R}}\mathrm{and}t_{2}=-\frac{2n+m}{d_{R}}$$

where *d_R_* is:

(5) $$d_{R}=\gcd(2n+m,2m+n)$$

Since the translation vector is parallel to the twist axis, it is equal to the length of twist circumference [25]:

(6) $$L=\left|\vec{T}\right|$$

On the other hand, for the length of twist graphene, we have:

(7) L=πzs

where *Z* is the diameter of the twist and *S* is the number of twists in twisted graphene. Hence, for any zigzag vector *C* (n, 0), we have:

(8) $$\vec{T}=\vec{a_{1}}-2\vec{a_{2}}=\sqrt{3}$$

Consequently, using various values of *S*, the diameter of twist can be obtained as:

(9) $$z=\frac{\sqrt{3}}{\pi s}$$

In the other words, it means that *Z* is proportional to:

(10) $$z\propto \frac{1}{2s}$$

**Figure 3:**
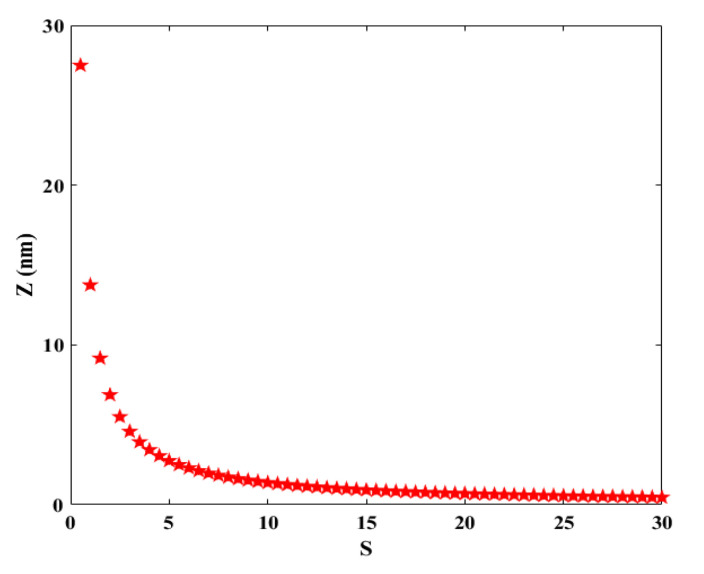
Z versus S in twisted graphene.

Figure 3 confirms the fact that when the number of twists is greater, the diameter of the twist is smaller, and vice versa.

Now, the electronic properties of graphene twist are surveyed. Using the Taylor expansion series, the E-K relation of graphene band structure can be given by [26]:

(11) $$E=\frac{3ta_{C-C}}{2}\sqrt{K_{x}{}^{2}+\beta^{2}}$$

Using some simplification, we have:

(12) $$E=E_{c}+\frac{E_{c}}{4\beta^{2}}K^{2}$$

where *Ec* is assumed as [27]:

(13) $$E_{c}=0.5t+\frac{3L^{2}}{2n^{2}}$$

Consequently, the amount of β is:

(14) $$\beta =\frac{tn^{2}a+3aL^{2}}{3tn^{2}a^{2}}$$

**Figure 4:**
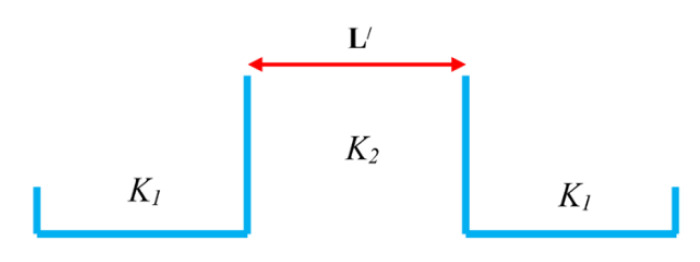
The 1D potential barrier of the proposed twisted graphene Schottky transistor.

Based on our proposed structure in Figure 2, the quantum tunneling effect for the Schottky barrier must be regarded. First, the transmission probability in two regions that wave vector changes from *K*_1_ to *K*_2_ should be calculated. Figure 4 indicates a sketch of Schottky contacts that *K*_1_ is the wave vector in the metallic region, *K*_2_ is the wave vector in the semiconducting region and *L′* is the length of the barrier. The transmission probability (*T* (*E*)) is given by [28]:

(15) $$T(E)={\left(1+{\left(\frac{K_{1}{}^{2}+K_{2}{}^{2}}{2K_{1}K_{2}}\right)}^{2}\sinh^{2}(K_{2}L^{\prime })\right)}^{-1}$$

By replacement of wave vectors in each region, we have:

(16) $$\begin{array}{l} T(E)=(1+{\left(\frac{\frac{4E}{3ta}(\frac{tn_{1}{}^{2}a+3a{\left(\pi sz\right)}^{2}}{3tn_{1}{}^{2}a^{2}})-2{\left(\frac{tn_{1}{}^{2}a+3a{\left(\pi sz\right)}^{2}}{3tn_{1}{}^{2}a^{2}}\right)}^{2}+\frac{4E}{3ta}(\frac{tn_{2}{}^{2}a+3a{\left(\pi sz\right)}^{2}}{3tn_{2}{}^{2}a^{2}})-2{\left(\frac{tn_{2}{}^{2}a+3a{\left(\pi sz\right)}^{2}}{3tn_{2}{}^{2}a^{2}}\right)}^{2}}{2\sqrt{\frac{4E}{3ta}(\frac{tn_{1}{}^{2}a+3a{\left(\pi sz\right)}^{2}}{3tn_{1}{}^{2}a^{2}})-2{\left(\frac{tn_{1}{}^{2}a+3a{\left(\pi sz\right)}^{2}}{3tn_{1}{}^{2}a^{2}}\right)}^{2}}\sqrt{\frac{4E}{3ta}(\frac{tn_{2}{}^{2}a+3a{\left(\pi sz\right)}^{2}}{3tn_{2}{}^{2}a^{2}})-2{\left(\frac{tn_{2}{}^{2}a+3a{\left(\pi sz\right)}^{2}}{3tn_{2}{}^{2}a^{2}}\right)}^{2}}}\right)}^{2}\times \\ \sinh^{2}(L^{\prime }\sqrt{\frac{4E}{3ta}(\frac{tn_{2}{}^{2}a+3a{\left(\pi sz\right)}^{2}}{3tn_{2}{}^{2}a^{2}})-2{\left(\frac{tn_{2}{}^{2}a+3a{\left(\pi sz\right)}^{2}}{3tn_{2}{}^{2}a^{2}}\right)}^{2}}){)}^{-1} \end{array}$$

The geometry effect in (16) can also be seen. Now, by applying a voltage *V* to the gate of Schottky transistor, the quantum current density (*J*) can be calculated as in [26,28]:

(17) $$J=\frac{e^{2}}{\pi \hbar }V_{applied}T_{\left(E\right)}$$

Consequently, by knowing that the current density definition in 1-D materials, Equation (17) is converted to:

(18) $$I=\frac{e^{2}}{\pi \hbar }L^{\prime }V_{applied}T_{\left(E\right)}$$

The applied voltage is given by:

(19) $$V_{app}=(((V_{GS}-V_{T})V_{DS}-\frac{V_{DS}{}^{2}}{2})+V_{T})$$

Where $V_{T}$ is the thermal voltage (26 mV). So, the *I-V* characteristic relation is obtained by (20):

(20) $$\begin{array}{l} I=\frac{e^{2}}{\pi \hbar }L^{\prime }(((V_{GS}-V_{T})V_{DS}-\frac{V_{DS}{}^{2}}{2})+V_{T})(1+{\left(\frac{\frac{4E}{3ta}(\frac{tn_{1}{}^{2}a+3a{\left(\pi sz\right)}^{2}}{3tn_{1}{}^{2}a^{2}})-2{\left(\frac{tn_{1}{}^{2}a+3a{\left(\pi sz\right)}^{2}}{3tn_{1}{}^{2}a^{2}}\right)}^{2}+\frac{4E}{3ta}(\frac{tn_{2}{}^{2}a+3a{\left(\pi sz\right)}^{2}}{3tn_{2}{}^{2}a^{2}})-2{\left(\frac{tn_{2}{}^{2}a+3a{\left(\pi sz\right)}^{2}}{3tn_{2}{}^{2}a^{2}}\right)}^{2}}{2\sqrt{\frac{4E}{3ta}(\frac{tn_{1}{}^{2}a+3a{\left(\pi sz\right)}^{2}}{3tn_{1}{}^{2}a^{2}})-2{\left(\frac{tn_{1}{}^{2}a+3a{\left(\pi sz\right)}^{2}}{3tn_{1}{}^{2}a^{2}}\right)}^{2}}\sqrt{\frac{4E}{3ta}(\frac{tn_{2}{}^{2}a+3a{\left(\pi sz\right)}^{2}}{3tn_{2}{}^{2}a^{2}})-2{\left(\frac{tn_{2}{}^{2}a+3a{\left(\pi sz\right)}^{2}}{3tn_{2}{}^{2}a^{2}}\right)}^{2}}}\right)}^{2}\times \\ \sinh^{2}(L^{\prime }\sqrt{\frac{4E}{3ta}(\frac{tn_{2}{}^{2}a+3a{\left(\pi sz\right)}^{2}}{3tn_{2}{}^{2}a^{2}})-2{\left(\frac{tn_{2}{}^{2}a+3a{\left(\pi sz\right)}^{2}}{3tn_{2}{}^{2}a^{2}}\right)}^{2}}){)}^{-1} \end{array}$$

## 3. Results and Discussion

**Figure 5:**
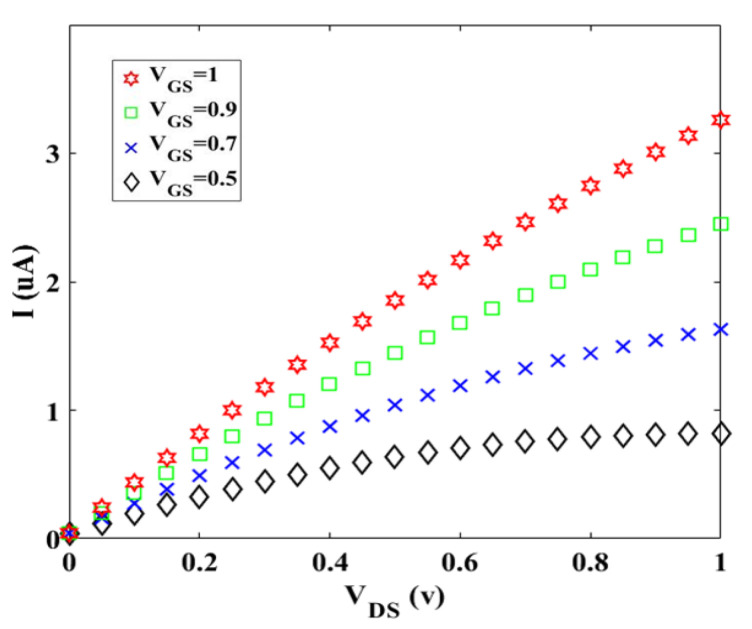
I-V characteristic for different values of VGS at room temperature.

In this part, the performance of twisted graphene-based Schottky transistor is studied and results, based on the analytical method using MATLAB software, are also discussed. Figure 5 represents the *I-V_DS_* at diverse values of *V_GS_*. It can be seen that the drain current rises substantially as the gate-source voltage is increased from 0.5 to 1 v which means that the gate-source voltage controls the current in the channel region (*I_D_*).

**Figure 6:**
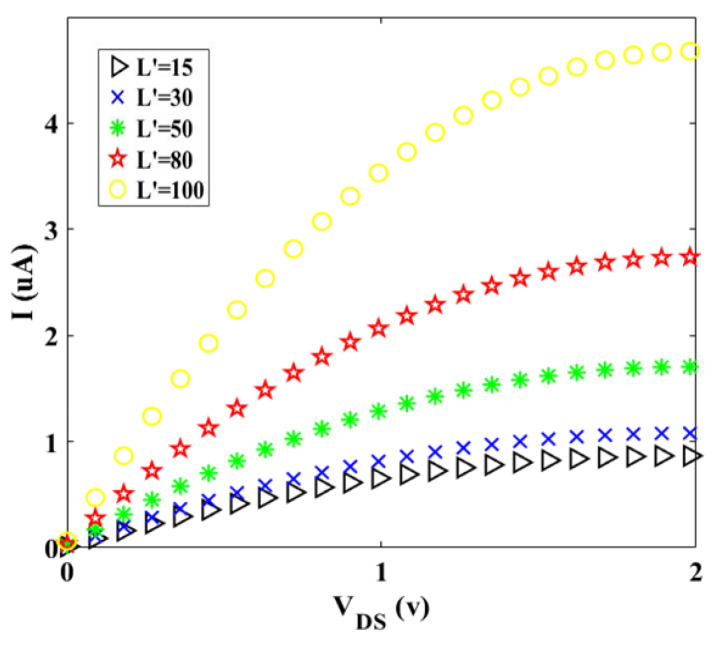
I-V characteristic for different values of channel length (in nm) at room temperature (*Z* = 10 and *S* = 5).

**Figure 7:**
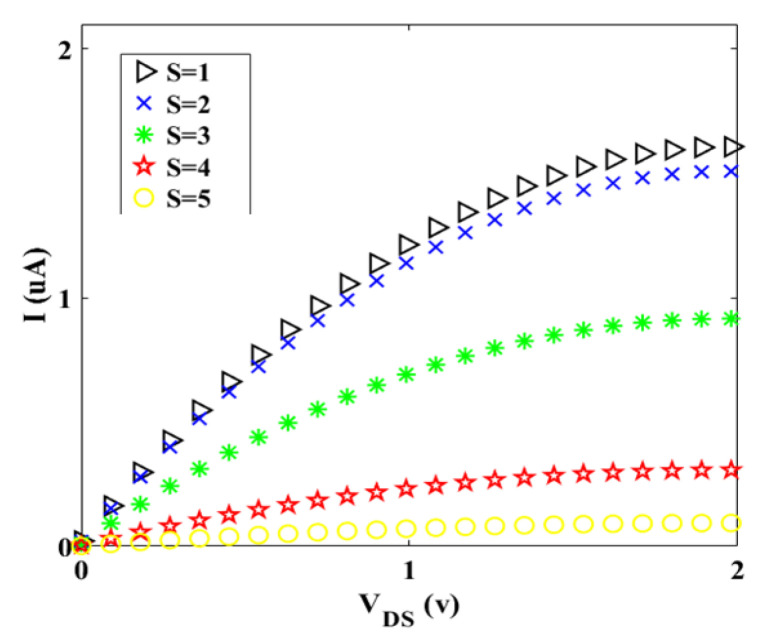
I-V characteristic for different values of S at room temperature (Z = 10).

**Figure 8:**
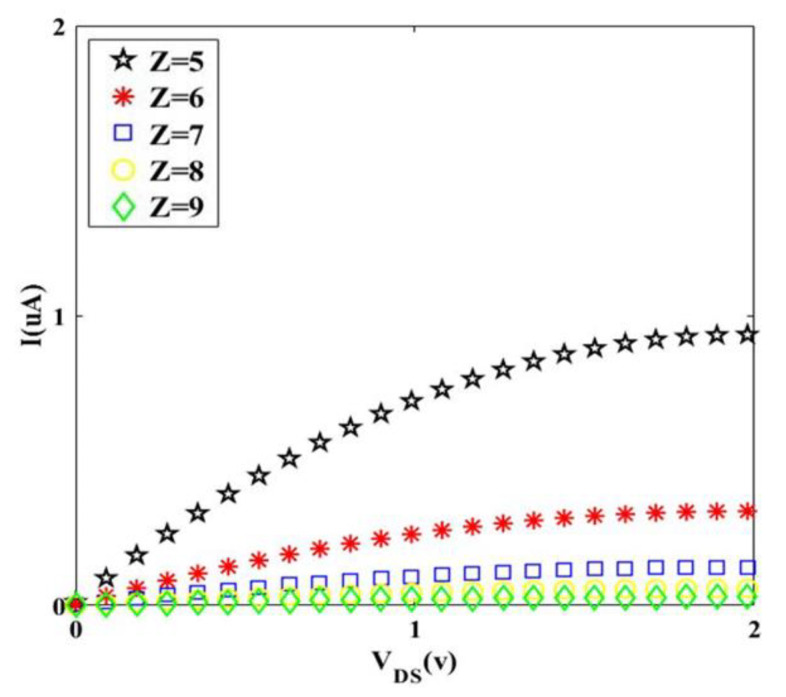
I-V characteristic for different values of Z at room temperature (S = 10).

Two factors, which are improving the gate electrostatic control and creating larger transconductance, are as the functions of Schottky transistors channel length [29]. Hence, the current-voltage characteristic for different values of channel length is plotted, as shown in Figure 6. For *L′* = 15 nm, it can be said that the electrons move easier than other values of *L′* and the tunneling effect not occurred in this case. In the other words, the electron passes through the barrier (moving directly), because the energy of the electron is more than the barrier (E≥eV). Figure 7 and Figure 8 indicate the geometry effect of twisted graphene on *I-V_DS_* characteristics. It can be seen from Figure 7 that by adding even one more twist in graphene twist, there is a dramatic descent in the initial slope of *I_D_* versus *V_DS_*.

It can be discerned from Figure 8 that the *I-V* characteristic is very sensitive, even to the addition of one nanometer to the diameter of graphene twist, which shows the importance of the *Z* role in twisted graphene Schottky transistor performance.

Although the *S* and *Z* based on Figure 3 have an inverse relation, nevertheless each of them has a reverse effect on transistor performance, which means that increasing in *S* or *Z* (separately) leads to the decrease in drain current that is controlled by the gate-source voltage. In the other words, small diameters of twisted graphene led to the transportation of electrons in one dimension, hence, the drain current increases [30]. In fact, the Fermi wavelength (*λ_F_*) represents that the wavefunctions of carriers that completely fill the diameter of the nanostructure. In the one-dimensional structure that the diameter is smaller than the *λ_F_*, the electrons in the 1D channel cannot screen the Coulomb potential from the gate and consequently, high current flows and great channel length modulation can be attained [31].

Also, the stress-strain of the twisted structure plays an important role in the variation of different properties [32,33]. Here, when the *S* is more, the amount of stress-strain of material is higher, and consequently, it leads to some defects in the structure. Defects caused the C-C bonds to break and new barriers to be generated. This phenomenon causes the perturbation of the drain current.

Hence, the drain current decreases. In comparison with Figure 7, in Figure 8, it can be said that even by the selection of larger *Z* (inset values of Figure 8) compared to amounts of *S* (inset values of Figure 7), the drain current is more for *S*, which means that the effect of *S* is higher than *Z* on Schottky transistor performance. This is due to the mechanical properties of twisted graphene structure, which means that if the twists (*S*) are not generated on graphene structure, the *Z* will not vary. In the other words, *Z* is a function of *S* (*f_(S)_ = Z*). Additionally, in the saturation region, that gradient of *I_D_-V_DS_* characteristic is zero, we have:

(21) $$V_{DS}(SAT)=(V_{GS}-V_{T})$$

Therefore, the *I_D_-V_GS_* characteristic for our proposed model can be written as:

(22) $$I=\frac{e^{2}}{\pi \hbar }L^{\prime }\left[{\left(\frac{V_{GS}-V_{T}}{2}\right)}^{2}+V_{T}\right]T_{\left(E\right)}$$

**Figure 9:**
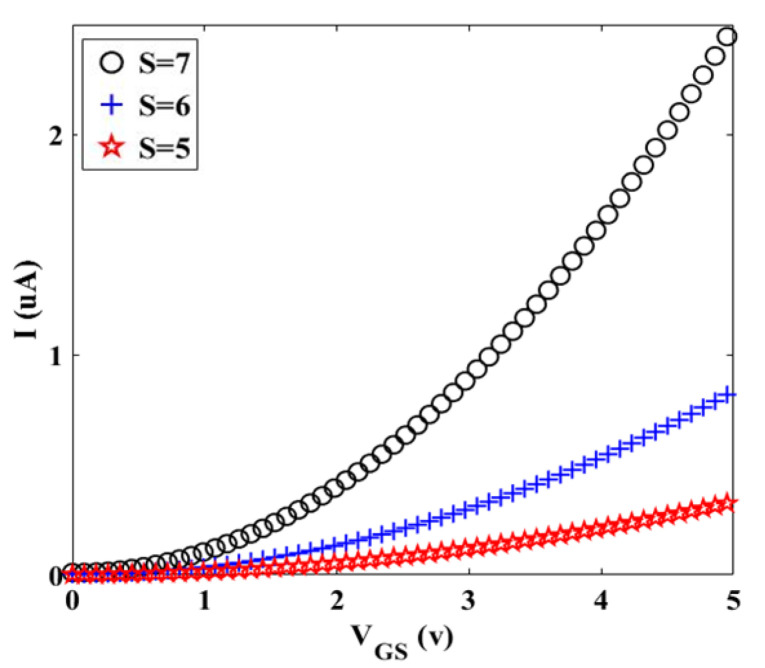
I- VGS characteristic for different values of S at room temperature (Z = 10).

**Figure 10:**
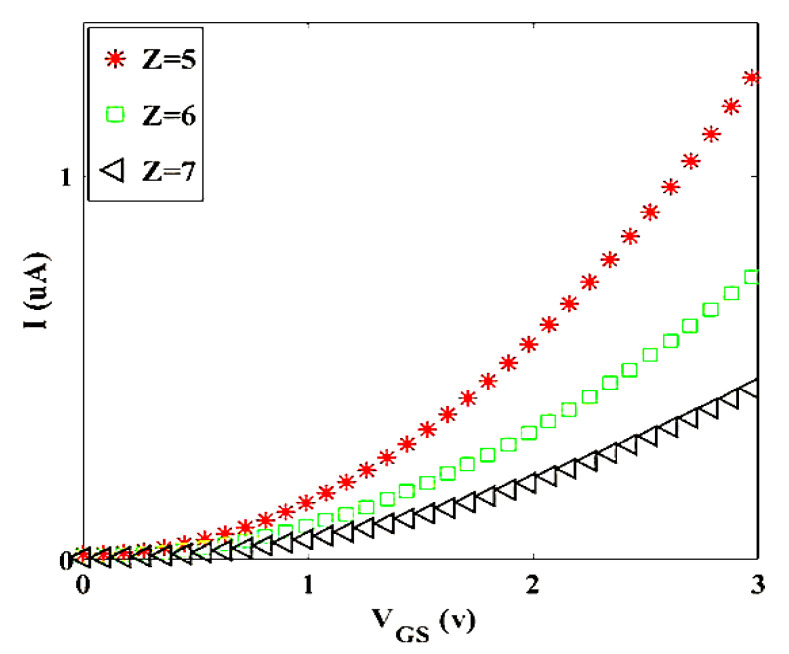
I- VGS characteristic for different values of Z at room temperature (S = 10).

Based on this exceptional equation, the threshold voltage of our transistor can be achieved. Figure 9 shows the *I_D_-V_GS_* characteristic for the diverse value of *S*. To investigate the *Z* effect on *I_D_-V_GS_* characteristic, Figure 10 is plotted for *S* = 10.

Figure 9 and Figure 10 illustrate the geometry effect of graphene twist on the threshold voltage of the proposed transistor that increment in the number of twists leads to decrease in threshold voltage of the transistor and it is very good for high-speed switching of transistor, because it causes less transistor power consumption. Figure 10 indicates that the diameter (*Z*) has an inverse relation on the threshold voltage of the transistor.

**Figure 11:**
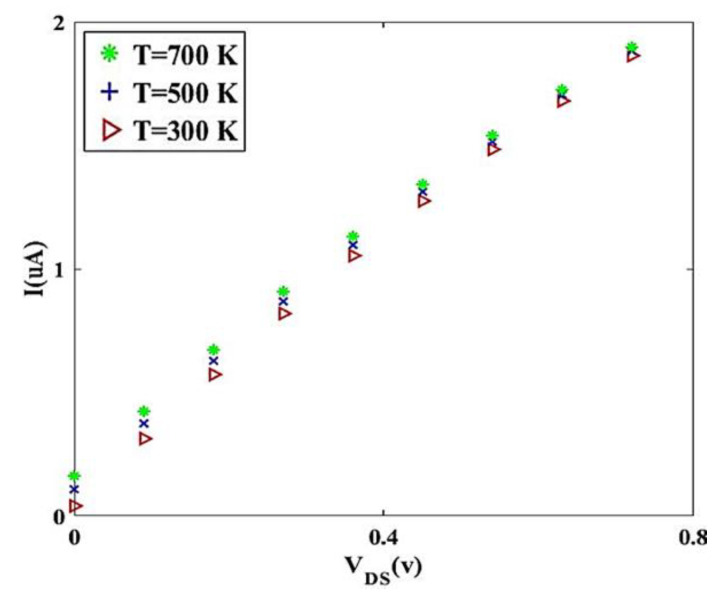
I-V characteristic for diverse values of temperature.

To compare Figure 9 and Figure 10, it can be noted that to optimize and improve the performance of twisted graphene Schottky transistor, it is better to choose twisted graphene with a smaller diameter. The temperature effect on transistor performance of twisted graphene is explored for three different amounts using thermal voltage (*V_T_*), as shown in Figure 11. Results indicate that increasing temperature leads to an increment in the mobility of electrons in the channel region of the twisted graphene Schottky transistor. Overall current in a real-world device can be lowered also by experimental non-idealities (e.g., contact resistances), which are typically not reflected in simulations.

**Figure 12:**
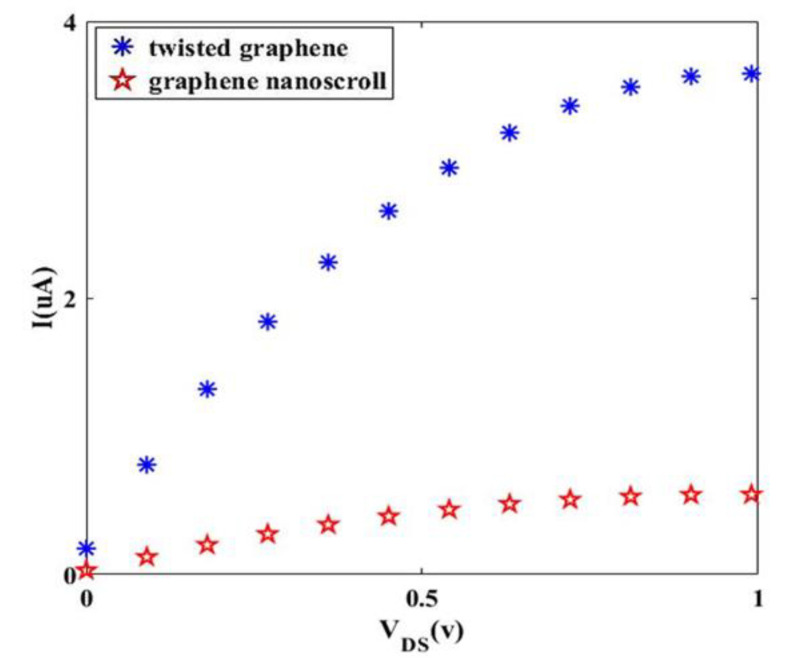
Comparison of the I-V characteristic of our proposed transistor and graphene nanoscroll-based Schottky transistor in the same channel length of 60 nm.

Finally, a comparative study of our modeled transistor and typical I-V characteristic of other transistors is depicted in Figure 12. In this figure, twisted graphene-based Schottky transistor and graphene nanoscroll-based Schottky transistor [26] in the same situation are considered. It can be noted that the channel length of both Schottky transistors is 60 nm, and they also have the same length of the circumference (*L*). As indicated in Figure 12, the proposed Schottky transistor has a larger drain current than the graphene nano scroll-based Schottky transistor, and it is a significant advantage for twisted graphene-based Schottky transistor. Additionally, Kosar et al. investigated the geometric, optical, and electronic responses of doped twisted graphene with alkalis and superalkalis in a density functional theory (DFT) study [34]. The differences between our research paper and this work are in the consideration of twisted graphene without any doped material, and also in the methodology of study that is based on tight binding calculations instead of DFT.

## 4. Conclusions

Twisted graphene with a novel structure is a promising material for nanoelectronic applications due to its remarkable electrical properties. In the presented work, a twisted graphene-based Schottky transistor is analytically modeled. The proposed structure with zigzag twisted graphene as metallic and semiconducting properties depends on its chirality numbers, which are assumed to be (19, 0) and (17, 0), respectively. The geometry of twisted graphene by translation (*T*) and chiral (*C*) vectors are explored and the relationship between diameter and number of twists for all zigzag vectors are calculated, considering the dispersion relation and tunneling effect the wave vector changes from *K*_1_ to *K*_2_ (metallic to semiconducting). By applying a voltage to the proposed structure, the *I-V* characteristics are studied. It is concluded that increasing the gate-source voltage leads to an increment in drain current. On the other hand, a reduction in diameter and number of twists can increase the drain current. The effect of twisted graphene geometry on the threshold voltage of Schottky transistor indicates that the great number of twists and small values of diameters leads to a low threshold voltage of the transistor. So, it is concluded that in order to promote the performance of twisted graphene-based Schottky transistor, twisted graphene with a small diameter and more number of twists is appropriate. Additionally, the temperature effect on transistor performance is explored, and observations show that temperature increases lead to an increment in the drain current. Finally, the proposed transistor is compared with similar research which represents the superiority of twisted graphene-based Schottky transistor over the graphene nanoscrolls-based Schottky transistor. Altogether, twisted graphene is a desirable nominee for transistor devices in integrated circuits as high-speed switching applications.

## Data Availability

The data presented in this study are available on reasonable request from the corresponding author.
